# Supplementary material for: pilE G-Quadruplex Is Recognized and Preferentially Bound but Not Processed by the MutL Endonuclease from Neisseria gonorrhoeae Mismatch Repair Pathway
Source: Int J Mol Sci. 2023 Mar 24;24(7):6167. doi: 10.3390/ijms24076167 (PMC10094033; doi:10.3390/ijms24076167)
Supplement: Supplementary file 1 [file ijms-24-06167-s001.zip › Supplementary Materials_final.pdf]

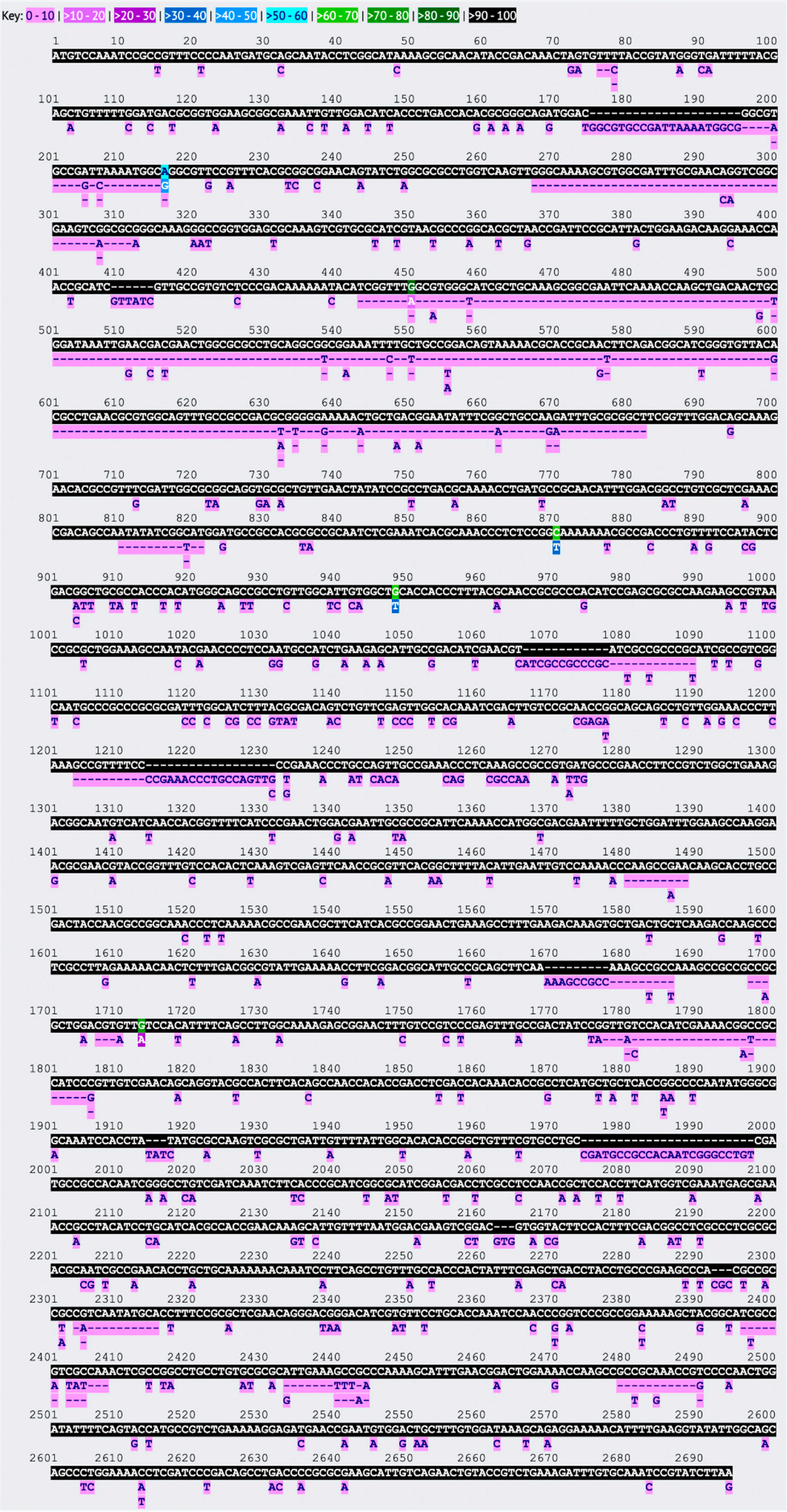
Key: 0 - 10 | >10 - 20 | >20 - 30 | >30 - 40 | >40 - 50 | >50 - 60 | >60 - 70 | >70 - 80 | >80 - 90 | >90 - 100

**Figure S1.** Polymorphism of *N. gonorrhoeae mutS.* Analysis of the NEIS2138 locus revealed the presence of 342 allelic variants with 867 polymorphic sites for 2697 positions. Numbers of the first nucleotide in the rows are indicated above of the sequences. The percentage of alleles that have certain nucleotide substitution(s) at each position of the coding strand are shaded with different colors: from the smallest (pink) to the highest (black). See key above the figure. Deletions are indicated as "-".

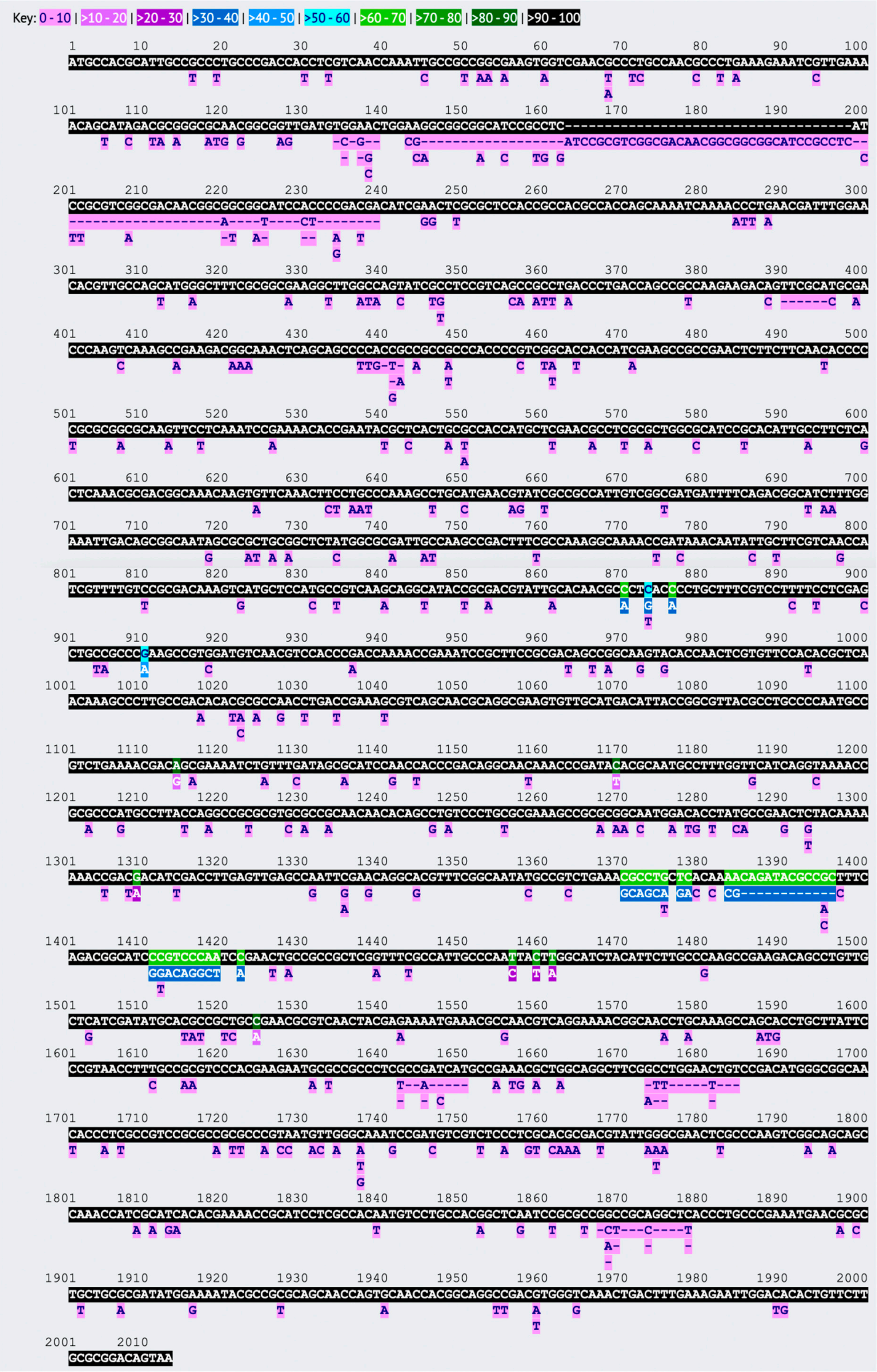
Key: 0 - 10 | >10 - 20 | >20 - 30 | >30 - 40 | >40 - 50 | >50 - 60 | >60 - 70 | >70 - 80 | >80 - 90 | >90 - 100

**Figure S2.** Polymorphism of *N. gonorrhoeae mutL*. Analysis of the NEIS2138 locus revealed the presence of 310 variants with 460 polymorphic sites for 2017 positions. Numbers of the first nucleotide in the rows are indicated above of the sequences.The percentage of alleles that have certain nucleotide substitution(s) at each position of the coding strand are shaded with different colors: from the smallest (pink) to the highest (black). See key above the figure. Deletions are indicated as "-".

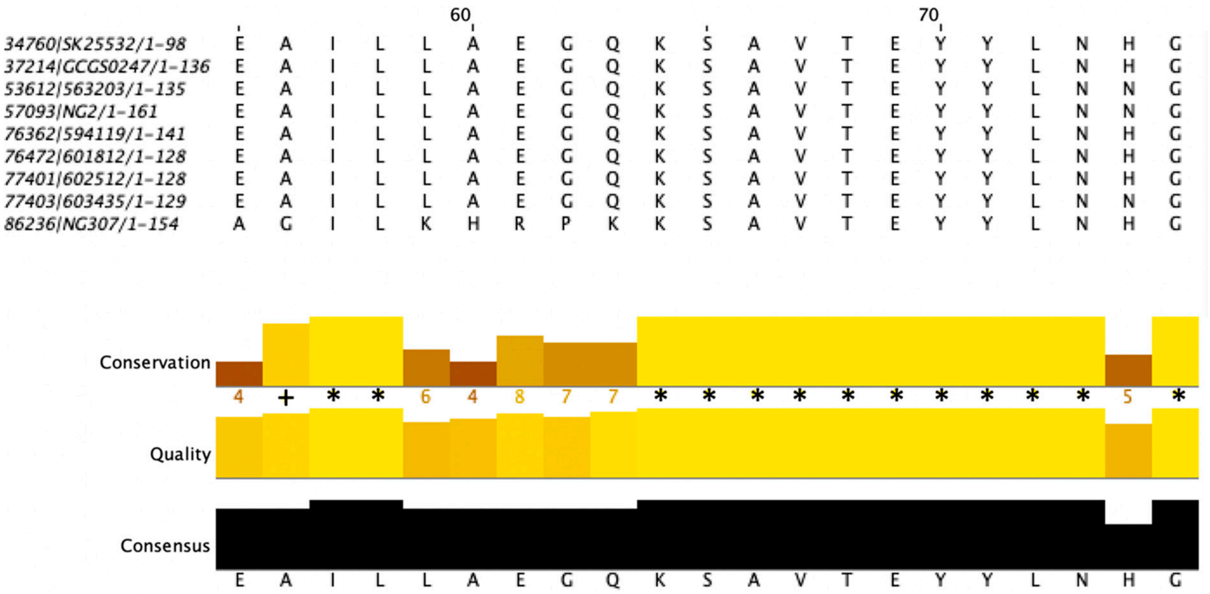


**Figure S3.** A part of an alignment of *N. gonorrhoeae* PilE amino acids from 3L group. The designations "*" correspond to completely conservative residues. The designations "+" indicate the small number of substitutions that do not affect the protein physico-chemical properties. Numerical indexes from 0 to 9 show the degree of identity in ascending order (0 corresponds to the most variable positions).

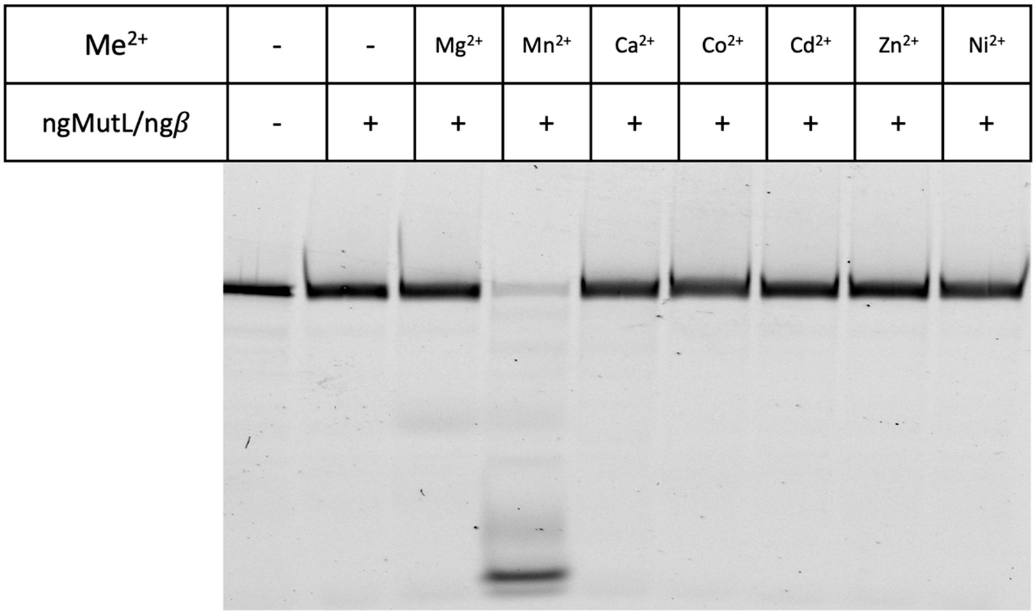


**Figure S4.** Effect of metal ions on ngMutL-induced hydrolysis of 76R/76M (10 nM). The reaction mixtures were incubated in the presence of 0.8 mM ATP, 5 mM metal ions ($Mg^{2+}$, $Mn^{2+}$, $Ca^{2+}$, $Co^{2+}$, $Cd^{2+}$, $Zn^{2+}$, $Ni^{2+}$) for 90 min at 37 °C, and then analyzed in a 12% polyacrylamide gel containing 7 M urea. 0.5 µM ngMutL (per dimer) was used in the presence of an equimolar amount of ngβ dimer.

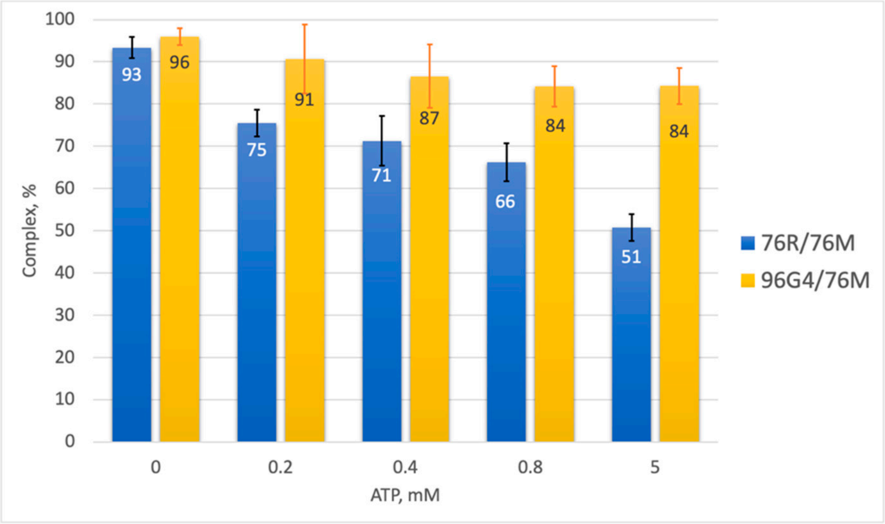


**Figure S5**. The effect of ATP on the ngMutL complex formation with 76R/76M (blue) and 95G4/76M (yellow) (20 nM concentration). The reaction mixtures (20 µL) were incubated for 10 min on ice with 500 nM ngMutL (per dimer) in 20 mM HEPES buffer (pH 8.0) containing 100 mM KCl, 0.5 mg/mL BSA, 5 mM $MgCl_2$ and 1 mM DTT.

Samples were analyzed by electrophoresis in a 6% polyacrylamide gel in TAE buffer for 2–3 h at 4 °C.
